# Fungal community assemblage of different soil compartments in mangrove ecosystem

**DOI:** 10.1038/s41598-017-09281-3

**Published:** 2017-08-17

**Authors:** Dinesh Sanka Loganathachetti, Anbu Poosakkannu, Sundararaman Muthuraman

**Affiliations:** 1 Department of Marine Biotechnology, Bharathidasan University, Tiruchirappalli, 620024 Tamil Nadu India; 2 University of Jyvaskyla, Department of Biological and Environmental Science, P.O. Box 35, FI-40014 Jyvaskyla, Finland

## Abstract

The fungal communities of different soil compartments in mangrove ecosystem are poorly studied. We sequenced the internal transcribed spacer (ITS) regions to characterize the fungal communities in *Avicennia marina* root-associated soils (rhizosphere and pneumatophore) and bulk soil compartments. The rhizosphere but not pneumatophore soil compartment had significantly lower fungal species richness than bulk soil. However, bulk soil fungal diversity (Shannon diversity index) was significantly higher than both pneumatophore and rhizosphere soil compartments. The different soil compartments significantly affected the fungal community composition. Pairwise sample analyses showed that bulk soil microbial community composition significantly different from rhizosphere and pneumatophore soil compartments. There was, however no significant difference observed between rhizosphere and pneumatophore soil fungal community composition and they shared relatively more OTUs between them. Further, there was a significant correlation observed between fungal community compositional changes and carbon or nitrogen availability of different soil compartments. These results suggest that few characteristics such as fungal richness and taxa abundance of rhizosphere and pneumatophore soil compartments were significantly different in mangrove ecosystem.

## Introduction

Mangroves generally grow in latitudes 32°N and 38°S falling in tropical and subtropical climate regions worldwide^1^. Mangroves are one of the most productive ecosystems that are known to actively participate in various bio-geo chemical processes^2, 3^. Several factors such as salinity fluctuation, inundation and anoxic nature of mangrove sediments create a stressful environment for the plants^1^. *Avicennia marina* is one of the major mangrove plants that generally grow in anoxic mangrove sediments of fringe or coastal mangroves^1^. *Avicennia marina* produce lateral roots as well as specialized root modifications called pneumatophores or aerial roots (pencil roots) in order to obtain physical stability in muddy mangrove sediments. Pneumatophores are produced from the lateral roots of the plants and grow vertically away from gravity. Pneumatophores carry out gaseous exchange, respiration and nutrient assimilation necessary for sustenance and growth of *A. marina* in prevailing atypical conditions of mangrove ecosystem^1, 4^. In addition, pneumatophores offer protection for *A. marina* from soil erosion^5^ and participate in biogeochemical cycles like carbon and nitrogen fixation^6–8^.

Previous studies investigated the diversity and functional characteristics of mangrove fungi on woody parts of trees, decomposing litter and sediments^9–17^. Atypical conditions of magrove roots may create a rich habitat, thus allowing the colonization of different microbial communities^5^. A very few studies have characterized the pneumatophore-associated (epi- and endosphere) fungi^9, 12, 14, 16, 17^. However, fungal diversity of lateral root attached soil (hereafter referred to as “rhizosphere”) in mangrove ecosystems is poorly studied^18–21^ and pneumatophore attached soil (hereafter referred to as “pneumatophore”) is virtually unknown. The comparative studies on fungal community of root-associated (pneumatophore and rhizosphere) soil compartments would help us in discerning the functional difference between them.

Similar to other ecosystems, fungi are predicted to play an important role in carbon and nitrogen recycling in mangrove ecosystems^22–25^. It has been shown that fungal isolates from mangrove ecosystems are able to synthesize the organic matter decomposing enzymes^26–29^. It is considered that the fungi initially begin the decomposition of plant materials in mangrove ecosystem^30^. The yeast and bacteria further complete the decomposition process^30^. The availability of carbon and nitrogen could influence the fungal community composition across the soil compartments. However, there is no information currently available pertaining to the correlation of microbial community composition to carbon and nitrogen availability in mangrove ecosystem.

Considering the functional specialty of pneumatophore roots in mangrove ecosystems, we hypothesized that root-associated soil compartments, rhizosphere and pneumatophore differ in their fungal richness (number of fungal taxa) and Shannon diversity (a combined measure of richness and the evenness of their abundance). We chose *A. marina* as focal tree due to its wide spread occurrence in fringe mangroves. In the present study, we tested the following predictions in mangrove ecosystem: (i) fungal richness and diversity are significantly different in rhizosphere, pneumatophore and bulk soil compartments (hereafter referred to as “bulk”), (ii) the selected soil compartments, bulk, pneumatophore and rhizosphere affect fungal community composition significantly and (iii) the availability carbon and nitrogen of different soil compartments affect a part of fungal community composition.

## Results

**Figure 1:**
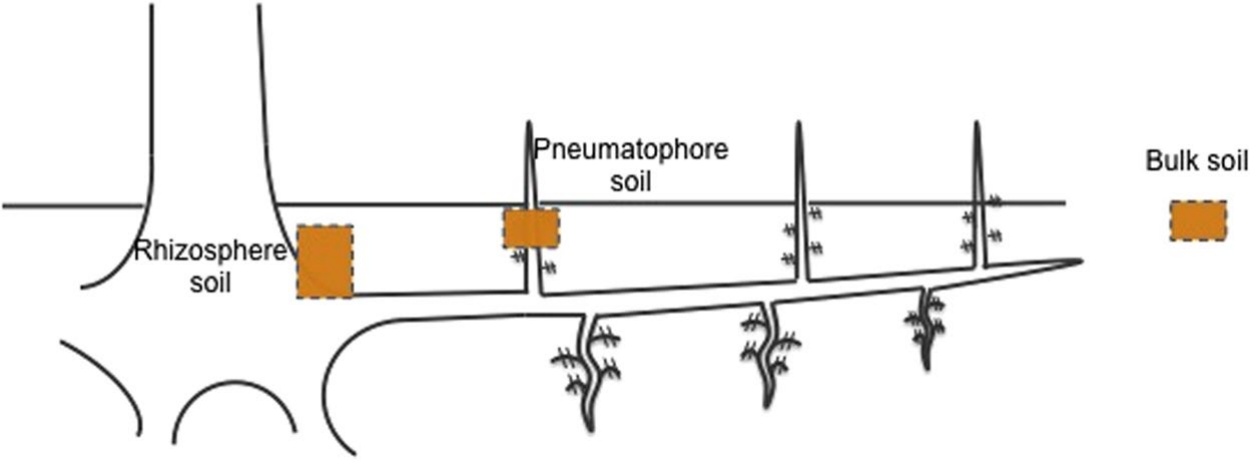
The schematic representation of sampling of three different soil compartments.

**Figure 2:**
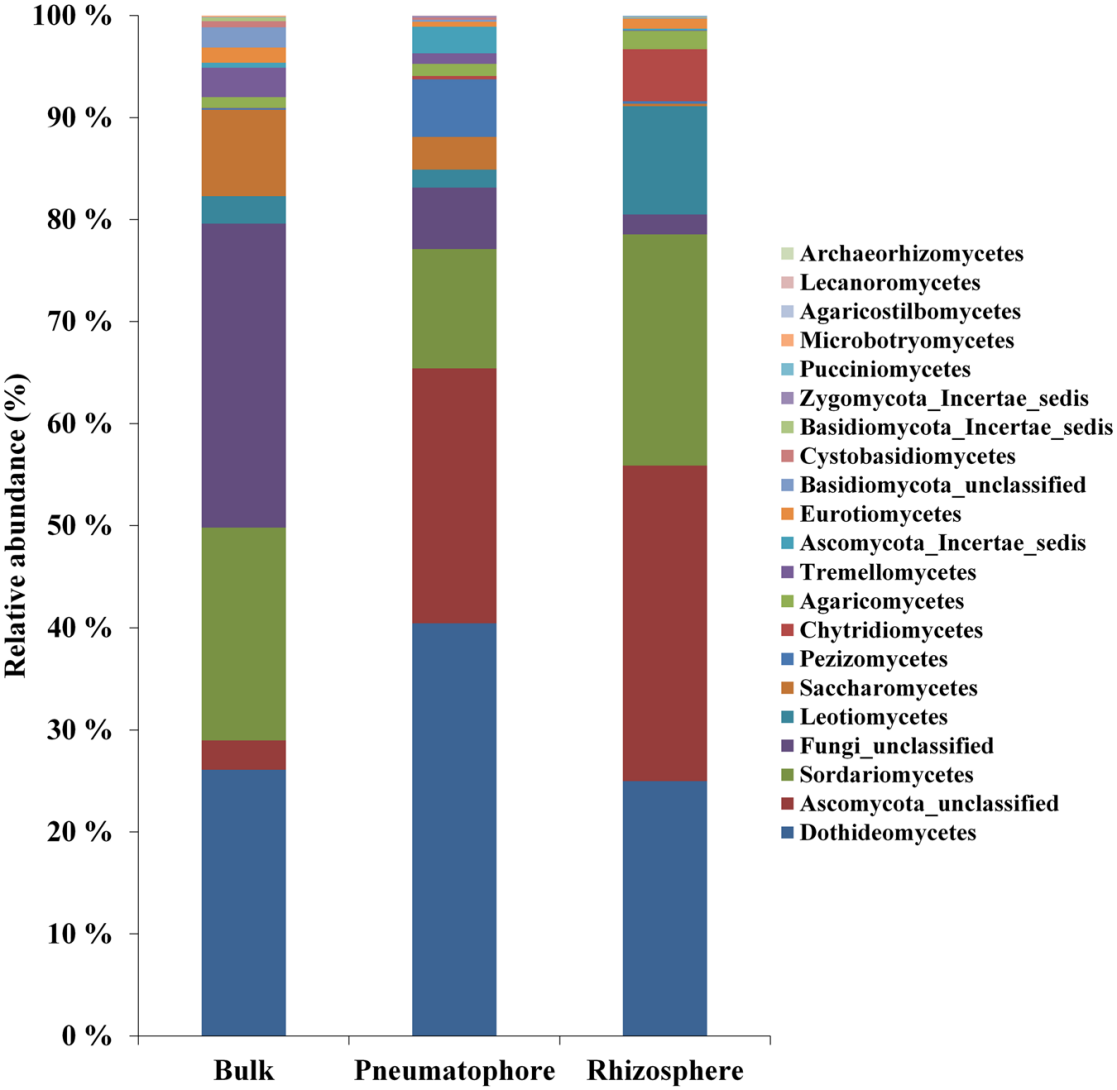
Class level distribution based on fungal ITS sequences of bulk, pneumatophore, and rhizosphere soil compartments. Values are mean of five replicates from each sample. OTUs were clustered at 97% similarity level.

We investigated fungal communities of bulk, rhizosphere, and pneumatophore soil compartments (Fig. 1) in a mangrove ecosystem. The root-associated soil compartments (rhizosphere and pneumatophore) were collected from *A. marina* trees. High-throughput sequencing of internal transcribed regions (ITS) was yielded 117, 688 good quality sequences (Supplementary file: Supplementary Table 1). The removal of rare OTUs (OTUs with less than five sequences) reduced the total number of sequences to 115 021 (Supplementary file: Supplementary Table 1). Good’s coverage (https://www.mothur.org/wiki/Coverage) scores were high across the compartments ranging from 0.97 to 0.99 indicating that the sequencing depth was adequate to dependably describe the fungal microbiome (Supplementary file: Supplementary Table 1). A total of 21 different fungal classes were identified across the three different compartments (Fig. 2). Out of which 20, 19 and 18 were found in bulk, rhizosphere and pneumatophore compartments, respectively.

### The fungal richness and diversity (Shannon) was affected by compartment

To gain insights into the effect of compartment on fungal richness and diversity (Shannon), we performed one way analysis of variance (ANOVA) with compartment as fixed factor. The analyses showed that both OTU richness and Shannon diversity index were significantly affected by compartments (Supplementary file: Supplementary Table 2).

**Figure 3:**
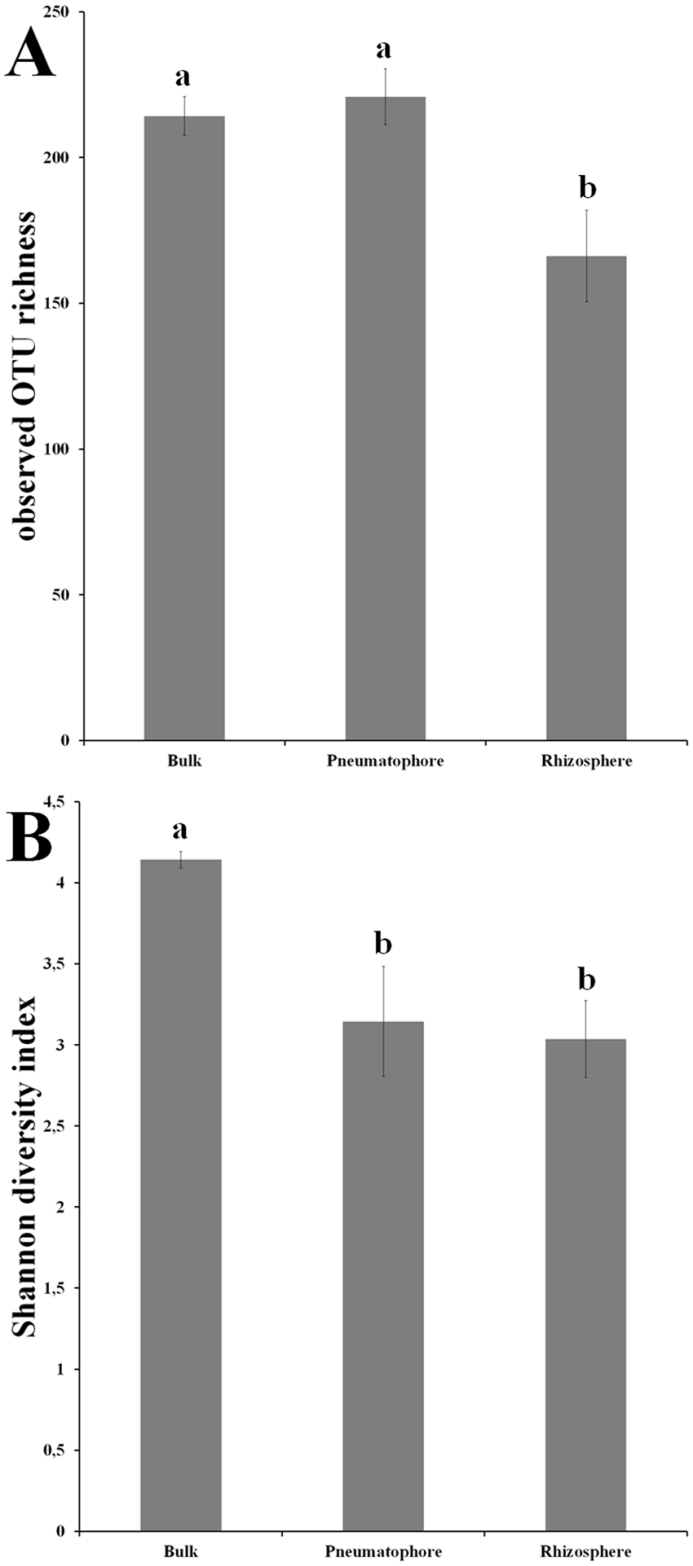
Observed OTU richness (**A**), and Shannon diversity index (**B**) of ITS fungal OTUs in bulk, pneumatophore and rhizosphere soil compartments. OTUs were clustered at 97% similarity level. The different alphabet denotes significance at 0.05 (ANOVA; Post-Hoc test: Tukey HSD).

Furthermore, in order to identify the significant difference between compartments, comparisons of observed OTU richness and Shannon diversity index were carried out using communities retrieved from bulk, rhizosphere and pneumatophore samples (Fig. 3). The pneumatophore accounted for the highest OTU richness followed by bulk. The rhizosphere accounted for the lowest OTU richness and it was significantly (p < 0.05) different from pneumatophore and bulk compartments (Fig. 3A). The bulk samples accounted for the highest Shannon diversity index and significantly (p < 0.05) different from pneumatophore and rhizosphere (Fig. 3B).

### Fungal community composition was affected by compartment

**Figure 4:**
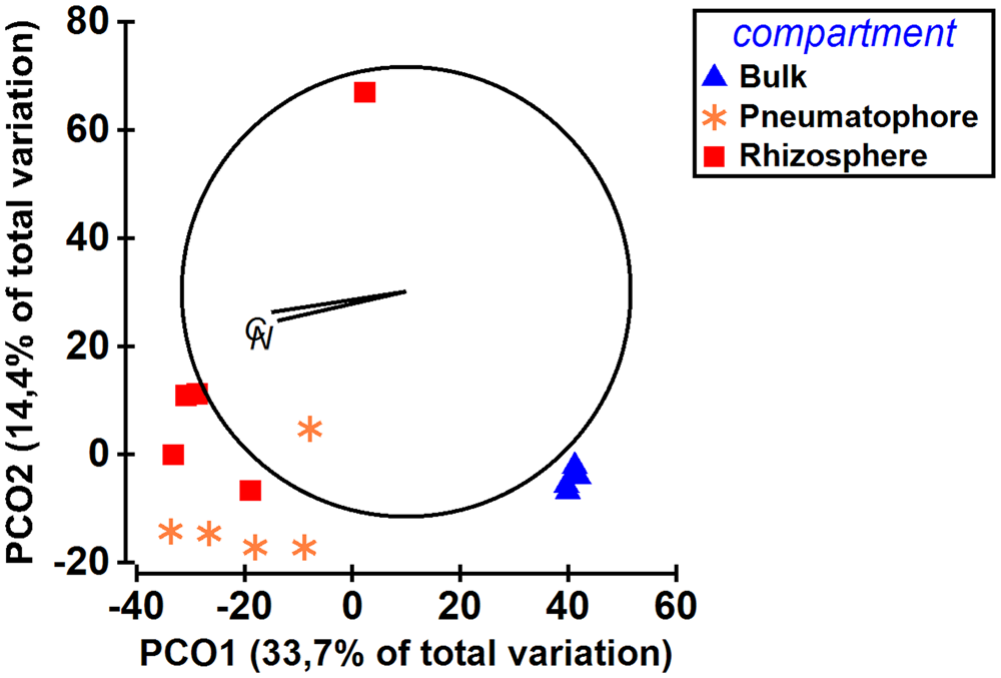
Clustering pattern of fungal community composition in bulk, pneumatophore and rhizosphere soil compartments. The C and N in the figure indicate the carbon and nitrogen content of the different soil compartments, respectively. The significant correlation was observed between fungal community composition and carbon or nitrogen content of the soil compartments. OTUs were clustered at 97% similarity level. The weighted and constrained principal-coordinate analysis (PCoA) based on Bray–Curtis dissimilarity on standardized and square-root transformed data was performed in PRIMER software v6.

To gain insights into the effect of compartment on fungal community composition, we performed unconstrained principal coordinate analysis (PCoA) to identify the clustering pattern of microbial communities. The bulk samples clustered separately from pneumatophore and rhizosphere compartments in global PCoA (Fig. 4). Permutational Multivariate Analysis of Variance (PERMANOVA) confirmed that the observed clustering pattern in PCoA was statistically significant (p < 0.05; Supplementary file: Supplementary Table 3). A total of 40.7% [(Factor sum of squares)/(total sum of squares) * 100] of the variations was explained by the compartment as fixed factor in PERMANOVA (Supplementary file: Supplementary Table 3).

We performed pairwise PCoAs of bulk Vs rhizosphere, bulk Vs pneumatophore, and rhizosphere Vs pneumatophore (Supplementary file: Supplementary figure 1). The PERMANOVA analyses showed that bulk Vs rhizosphere and, bulk Vs pneumatophore clustering patterns were statistically significant (p < 0.05; Supplementary file: Supplementary Table 4). But, rhizosphere Vs pneumatophore clustering pattern was not significantly different (p > 0.05; Supplementary file: Supplementary Table 4).

We performed Kruskal–Wallis test with log transformed (log [X + 1]) relative abundance data to identify the OTUs that are responsible for community separation in different compartments. To exclude rare OTUs, we included OTUs with at least 1% abundance in minimum of three samples, which resulted in 25 fungal OTUs. There were 13 and 11 OTUs that displayed significant (p < 0.05) difference between bulk Vs pneumatophore and bulk Vs rhizosphere compartments, respectively (Supplementary file: Supplementary Table 5). There was no significant difference observed in any OTUs between pneumatophore and rhizosphere soil compartments (Supplementary file: Supplementary Table 5).

### The higher order fungal taxa were affected by compartment

**Figure 5:**
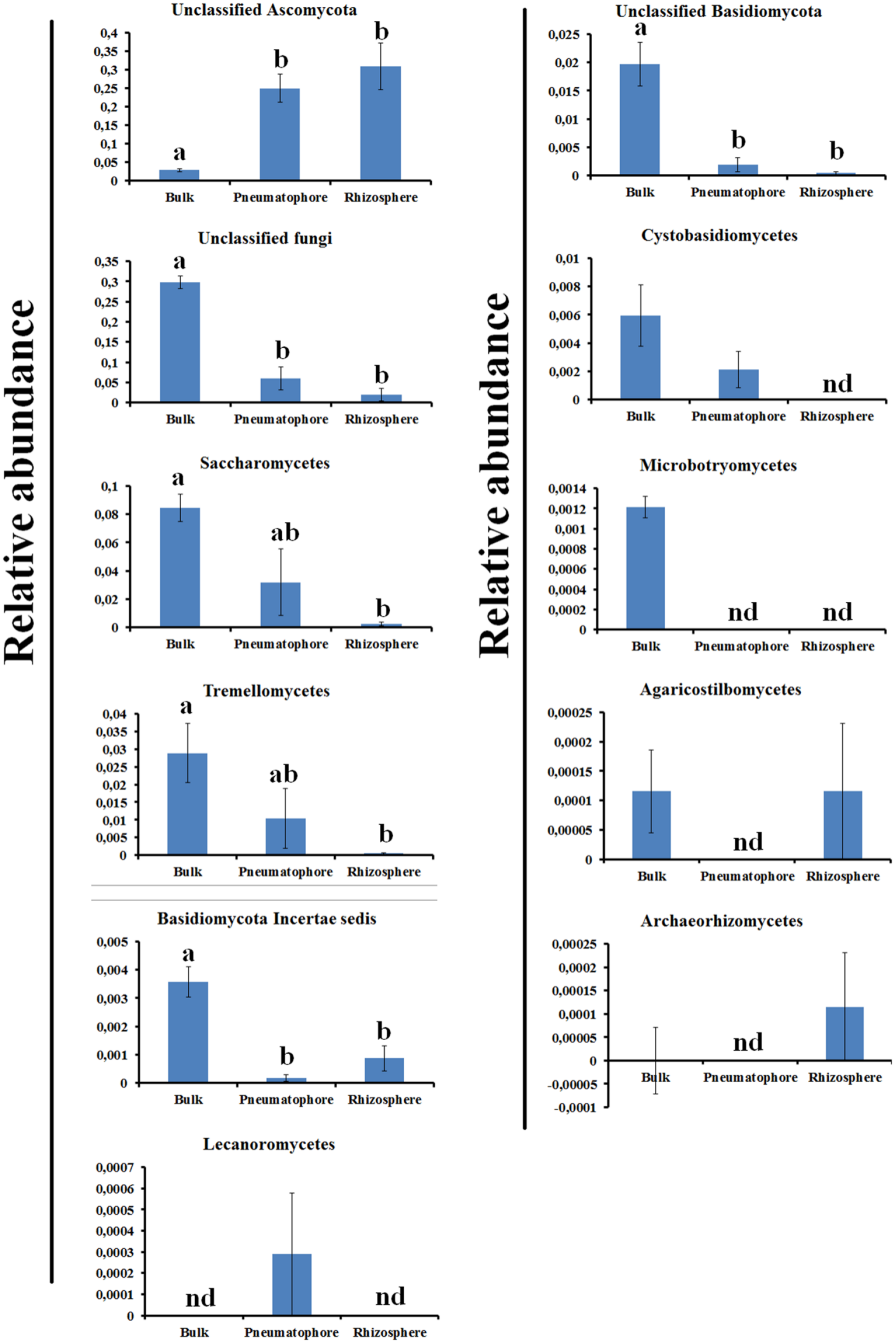
Differentially abundant fungal classes across the compartments. OTUs were clustered at 97% similarity level. The different alphabet (a,b and ab) denotes significance at 0.05 (Bonferroni correction). The letter “nd” denotes “not discovered”.

We applied linear statistics to identify the fungal classes that differed across the compartments. Out of 21 total classes identified, 11 of them were highly enriched in one or two compartments (Fig. 5). For instance, unclassified Ascomycota was highly enriched (p < 0.05) in rhizosphere and pneumatophore compartments compared to bulk. In contrast, Saccharomycetes was highly enriched (p < 0.05) in bulk and pneumatophore compartments compared to rhizosphere.

### Root-associated compartments shared relatively more OTUs

**Figure 6:**
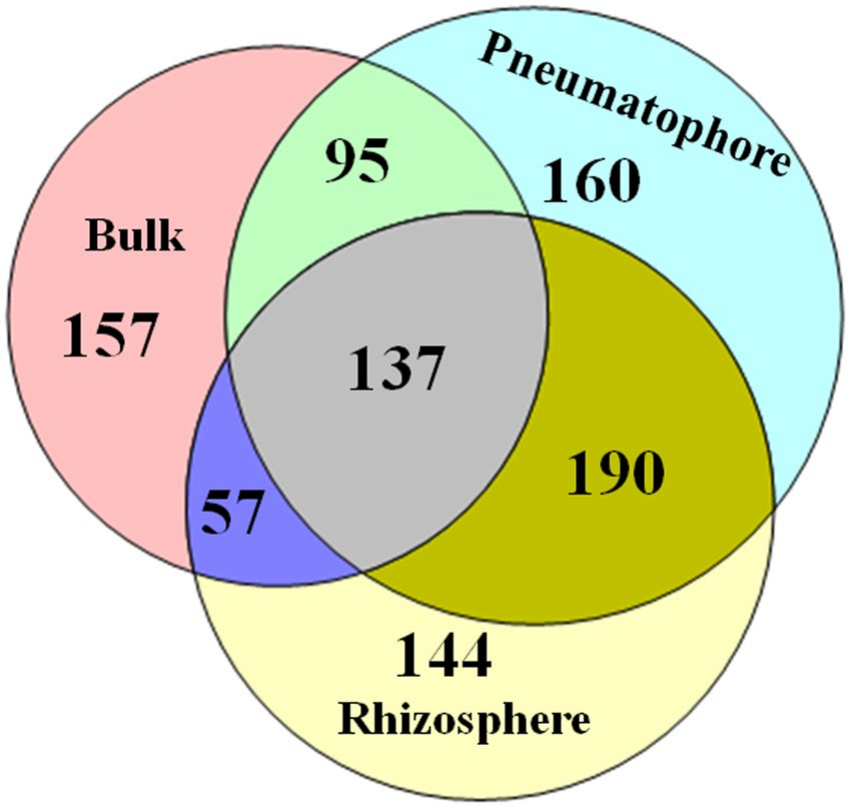
Venn diagram showing the number of shared and unique fungal OTUs (ITS region) in bulk, rhizosphere and pneumatophore compartments. OTUs were clustered at 97% similarity level. Venn diagrams were generated in Mothur v.1.35.0 and modified using Venn diagram plotter software.

A total of 446, 582, and 528 OTUs were present in bulk, pneumatophore and rhizosphere compartments, respectively (Fig. 6). A total of 137 OTUs were shared between bulk, pneumatophore and rhizosphere compartments (Fig. 6). A total of 23% of OTUs were shared between bulk and pneumatophore compartments. The rhizosphere and pneumatophore compartments shared relatively higher percentage OTUs (29%) followed by bulk and pneumatophore compartments (23%). The bulk and rhizosphere compartments shared 20% of OTUs between them.

### Carbon and nitrogen content of different compartments determined a part of fungal community diversity (Shannon) and composition

The carbon and nitrogen content of pneumatophore were significantly (p < 0.05) higher than bulk (Supplementary file: Supplementary Table 2). In contrast, the carbon and nitrogen content of rhizosphere was comparable to the pneumatophore and bulk.

Pearson correlation analysis showed that no significant (p > 0.05) correlation observed between species richness and carbon or nitrogen content of different compartments (Supplementary file: Supplementary Table 6). However, there was a significant (p < 0.01) correlation observed between Shannon diversity and carbon or nitrogen content of different compartments (Supplementary file: Supplementary Table 6 and Supplementary Figure 3). Similarly, distance based linear model analysis (DistLM) showed that significant correlation between fungal community composition with carbon or nitrogen content of different compartments (p < 0.05; Table 1). The nitrogen and carbon content of the soil compartments explained 15% and 16% of the variation in fungal community composition, respectively (Table 1).

**Table 1:** Distance based linear model statistical test (DistLM) for correlation between fungal community composition and nitrogen and carbon content in bulk, rhizosphere and pneumatophore soil compartments.

Source of variation	SS (trace)	Pseudo-F	p-value	Proportions
Nitrogen	6207.7	2.307	0.005	0.15072
Carbon	6626.8	2.4927	0.008	0.16089
Total	41187			

## Discussion

The fungal diversity and community composition in different soil compartments of mangrove ecosystem is poorly studied^21^. To our knowledge, this is the first study to characterize the pneumatophore soil fungal communities in mangrove ecosystem. Furthermore, we compared the fungal communities from bulk, pneumatophore and rhizosphere compartments. Our results showed that the different soil compartments of *A. marina* affect the fungal richness, diversity (Shannon) and community composition in the present mangrove ecosystem.

We hypothesized that fungal richness and diversity were significantly different in rhizosphere, pneumatophore and bulk compartments. Our results were not consistent with our hypothesis as bulk fungal OTU richness was higher than the rhizosphere compartment. In contrast, the pneumatophore fungal richness was comparable to bulk. The difference in sampling depth and more anaerobic condition in rhizosphere samples could be the potential reason for difference in lower richness of rhizosphere samples compared to pneumatophore. However, Shannon diversity of the bulk was higher than the both pneumatophore and rhizosphere compartments. The bulk fungi were more diverse than rhizosphere soil in other ecosystems^31, 32^. In contrast, it has been shown that fungal diversity was higher in rhizosphere than bulk in similar mangrove environment^21^. The difference in sampling depth of rhizosphere samples between the present (30 cm) and earlier (10 cm) studies^21^ could be the possible reason for contrasting results.

The fungal community composition of bulk was significantly different from root-associated soil compartments (pneumatophore and rhizosphere soils). This is consistent with fungal richness and diversity observations of this study. In addition, the root-associated compartments shared relatively more OTUs between them than individually with bulk. It is well known that the plant compounds released by roots play a major role in selecting the root-associated soil fungal communities^32–34^. In this study, the clustering pattern of different soil compartments showed that within sample variation was more pronounced in root-associated soil compartments than bulk. In contrast to our study, it has been shown that within sample variation was higher in bulk than rhizosphere in another mangrove ecosystem^21^. The difference in study site and methods (whole metagenomic sequencing Vs targeted amplicon sequencing) could be the potential reason for the dissimilarity between present and earlier^21^ studies.

The rhizo-deposited photosynthate and other exudates are important source of carbon and nitrogen for the microorganisms in the neighborhood of roots^34^. In this study, carbon and nitrogen content of root-associated samples were higher than bulk. Interestingly, only a fraction of variation in community composition was explained by carbon and nitrogen content of soil compartments. Also, negative correlation was observed between Shannon diversity and carbon or nitrogen content of the soil compartments. The carbon and nitrogen availability is considered to be one of the determinants of microbial communities in ecosystems^35, 36^.

The Dikarya (Ascomycota and Basidiomycota) were the most abundant phyla in this study, which is consistent with previous works on marine surface^37–39^ and mangrove ecosystems^15, 21, 40^. In this study, Dothideomycetes was the most abundant class (31% of total sequences) across the samples. In contrast, Dothideomycetes has been reported as a low abundant class of *A. marina* rhizosphere and sediments^21^. In this study, Saccharomycetes abundance was high in bulk compared to rhizosphere. Saccharomycetes has been reported as one of the major abundant fungal classes in anoxic mangrove sediment^15^. In contrast, Saccharomycetes is shown to be highly abundant in rhizosphere compared to bulk^21^. Our results and earlier study^21^ showed that the site-specific characteristics are the main reason for selecting the major root-associated soil fungal communities in mangrove ecosystems.

In this study, *Amorosia littoralis* (OTU1009) was one of the most abundant species in pneumatophore and rhizosphere samples compared to bulk. *Amorosia littoralis* has been reported as endophytic fungi of different mangrove trees including *A. marina* ^41^. Another major OTU (OTU0486) of this study, classified as *Debaryomyces nepalensis* was abundant in bulk compared to pneumatophore and rhizosphere soils. *Debaryomyces nepalensis* is a well-known salinity tolerant yeast species reported in mangrove environments^42, 43^. The higher presence of *D. napelensis* in the present mangrove ecosystem is justified due to higher salinity irrespective of seasonal difference^44^.

In conclusion, few characteristics such as species richness and taxa abundance were significantly different between pneumatophore and rhizosphere soils in this study. Most of the characteristics of the root-associated soil fungal communities (pneumatophore and rhizosphere) were significantly different from bulk soil. This indicates the possible effect of pneumatophore on surrounding soil and could be termed as “pneumatosphere” similar to rhizosphere for main roots. Further targeted studies are needed to understand the functional difference of pneumatosphere and rhizosphere soil fungal communities.

## Materials and Methods

### Site description and Sample collection

The sampling site is located at the edge of Vellar River and Bay of Bengal (11.49° N 79.76° W). The sampling was conducted during low tide season on 6th December 2014. The samples were collected from five trees separated by 5 to 12 meters distance apart. The soil samples were collected using poly vinyl carbonate (PVC) corer having 5 cm width. All the soil samples are composite of three corers per tree. The samples were collected from the following depths, bulk (0–10 cm), pneumatophore (0–10 cm) and rhizosphere (30 cm below the ground level). The bulk samples were collected 2–3 meters away from base of the tree, where influence of root is not present. Pneumatophore attached soil samples were collected from uprooted pneumatophores with the help of corer. Rhizosphere attached soil samples were collected vertically from base of the tree so as to avoid the collection of pneumatophore.

### Carbon and nitrogen content analyses

Carbon and nitrogen content analyses were carried out using C-H-N analyzer (http://www.sticindia.com/saif_instruments.html#chanl) at Sophisticated Test & Instrumentation Centre (Cochin University of Science and Technology), Cochin, India.

### Soil DNA extraction and high-throughput sequencing

The DNA was extracted from about 200 mg of soil using Power Soil DNA isolation kit (Mobio, USA) following manufacturer’s protocol with minor modification in the initial lysis step by vortexing at high–speed for 25–30 minutes. The soil DNA was extracted from all the samples within 24 hours of sampling and stored in −80° C. The PCR reaction and ion torrent sequencing was carried out as described in ref. 45. Briefly, we used fITS7 (5′-GTGARTCATCGAATCTTTG-3′) and ITS4 (5′-TCCTCCGCTTATTGATATGC-3′) in order to overcome the long amplicon bias^46^. The PCR amplifications were performed as follows: one cycle of 5 minutes at 94 °C, followed by 30 cycles of 30 seconds at 94 °C, 45 seconds at 53 °C, and 90 seconds at 72 °C, followed by one cycle of 7 minutes at 72 °C. We used M13 system based library preparation^47^. The sequencing was carried out using Ion PGM Sequencing 400 Hi-Q Kit (Ion 314 chips; Life Technologies, Thermo Fisher Scientific, Waltham, Massachusetts, USA) following manufacturer’s instructions.

### Bioinformatics

The bioinformatics analyses were carried as described in ref. 45 using Mothur software (v. 1. 35. 0)^48^. In brief, the parameters used were minlength = 200, maxambigs = 0, maxhomop = 8, qwindowaverage = 25, qwindowsize = 50 and bdiffs = 1. We used ITSx 1.0.7 software to extract the ITS2 region for better clustering of the sequences. Chimeric reads were removed using UCHIME 4.2 software (http://drive5.com/uchime). Fungal ITS sequences were clustered at 97% sequence similarity thresholds using CD-HIT 4.6.1 (cd-hit.org). Taxonomic identities were assigned to the sequences using the UNITE + INSD datasets (http://www.mothur.org/wiki/UNITE_ITS_database). Further, we removed the rare OTUs (less than five sequences) and normalized to their lowest number (3 464) of sequences for the further downstream analyses. OTU richness, Shannon diversity indices and venn diagram were calculated in mothur (v. 1. 35. 0)^48^.

### Statistical analyses

The clustering pattern of fungal community composition of different soil compartments was visualized using unconstrained principal coordinate analysis (PCoAs) and the statistical significance of clustering pattern was estimated by Permutational Multivariate Analysis of Variance (PERMANOVA)^49^. To test the correlation between fungal community composition and carbon and nitrogen content of different soil compartments, we performed distance based linear model (DistLM) statistical analyses. All the above statistical analyses were performed in PRIMER software V6^50^. We used generalized linear statistical model analysis to test the difference in carbon and nitrogen content of different soil compartments after testing the homogeneity of variances using the SPSS software (IBM SPSS v24).

### Data availability statement

Raw sequence data of all the 15 samples were submitted to sequence read archive (SRA accession number: SRP102307).

## Electronic supplementary material

Supplementary file
