# Supplementary material for: Fungal community assemblage of different soil compartments in mangrove ecosystem
Source: Sci Rep. 2017 Aug 17;7:8560. doi: 10.1038/s41598-017-09281-3 (PMC5561109; doi:10.1038/s41598-017-09281-3)
Supplement: Supplementary file 1 — Supplementary file [file 41598_2017_9281_MOESM1_ESM.doc]

**Fungal community assemblage of different soil compartments in mangrove ecosystem**

**Dinesh Sanka Loganathachetti^1^, Anbu Poosakkannu^2^*****, Sundararaman Muthuraman^1^***

1) Department of Marine Biotechnology, Bharathidasan University, Tiruchirappalli, 620024, Tamil Nadu, India.

2) Department of Biological and Environmental Science, PO Box 35, FI-40014 University of Jyväskylä, Finland.

*Corresponding authors

Sundararaman Muthuraman

Department of Marine Biotechnology

Bharathidasan University

Tiruchirappalli, 620024

Tamil Nadu, India

Tel: +91 - 431- 2407084; Fax: +91-431-2407082

Email: Sundar@bdu.ac.in

Anbu Poosakkannu

Department of Biological and Environmental Science

PO Box 35, FI-40014 University of Jyväskylä

Finland

Tel: +358 (0) 466137555; Fax: +358 (0) 14 617 239

Email: [anbu.a.poosakkannu@jyu.fi](mailto:anbu.a.poosakkannu@jyu.fi)


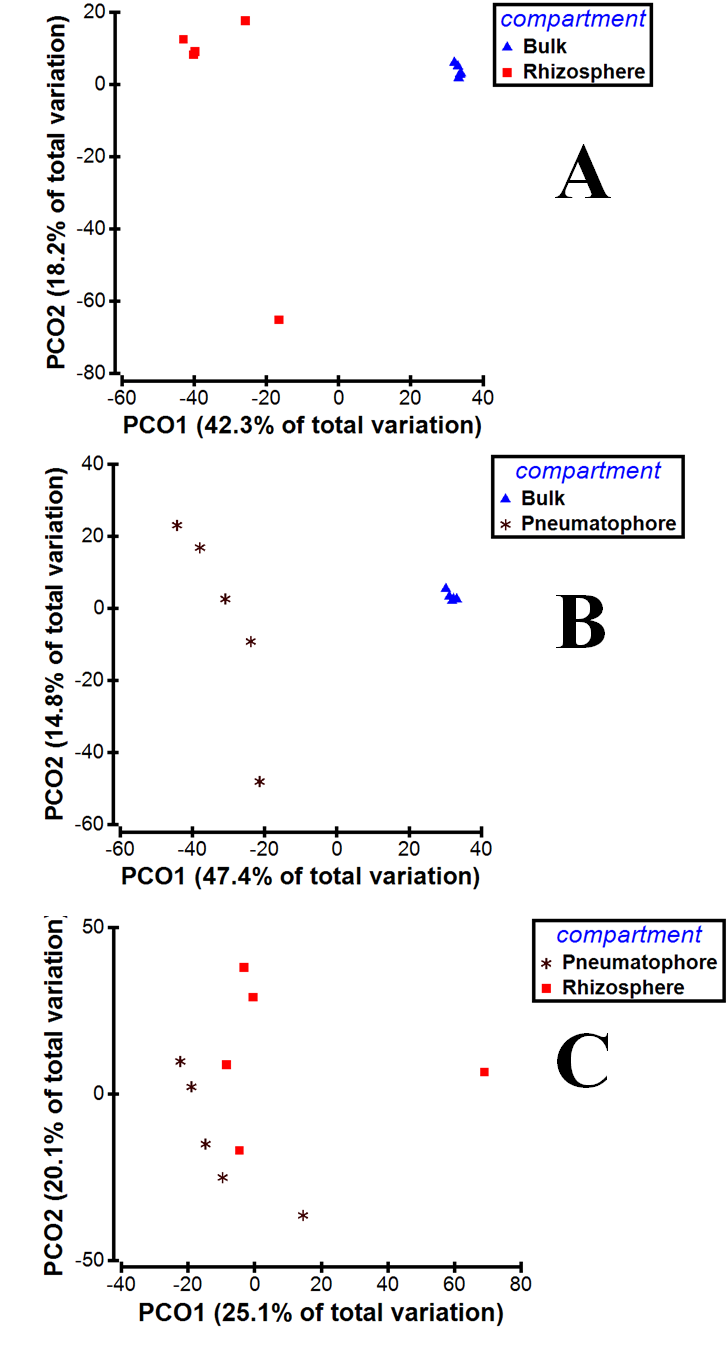


Supplementary figure 1. Clustering pattern of fungal community composition in pairwise comparisons of bulk Vs rhizosphere soil (A), bulk Vs pneumatophore (B), and rhizosphere Vs pneumatophore (C). OTUs were clustered at 97% similarity level. The weighted and constrained principal-coordinate analysis (PCoA) based on Bray–Curtis dissimilarity on standardized and square-root transformed data was performed in PRIMER software v6.


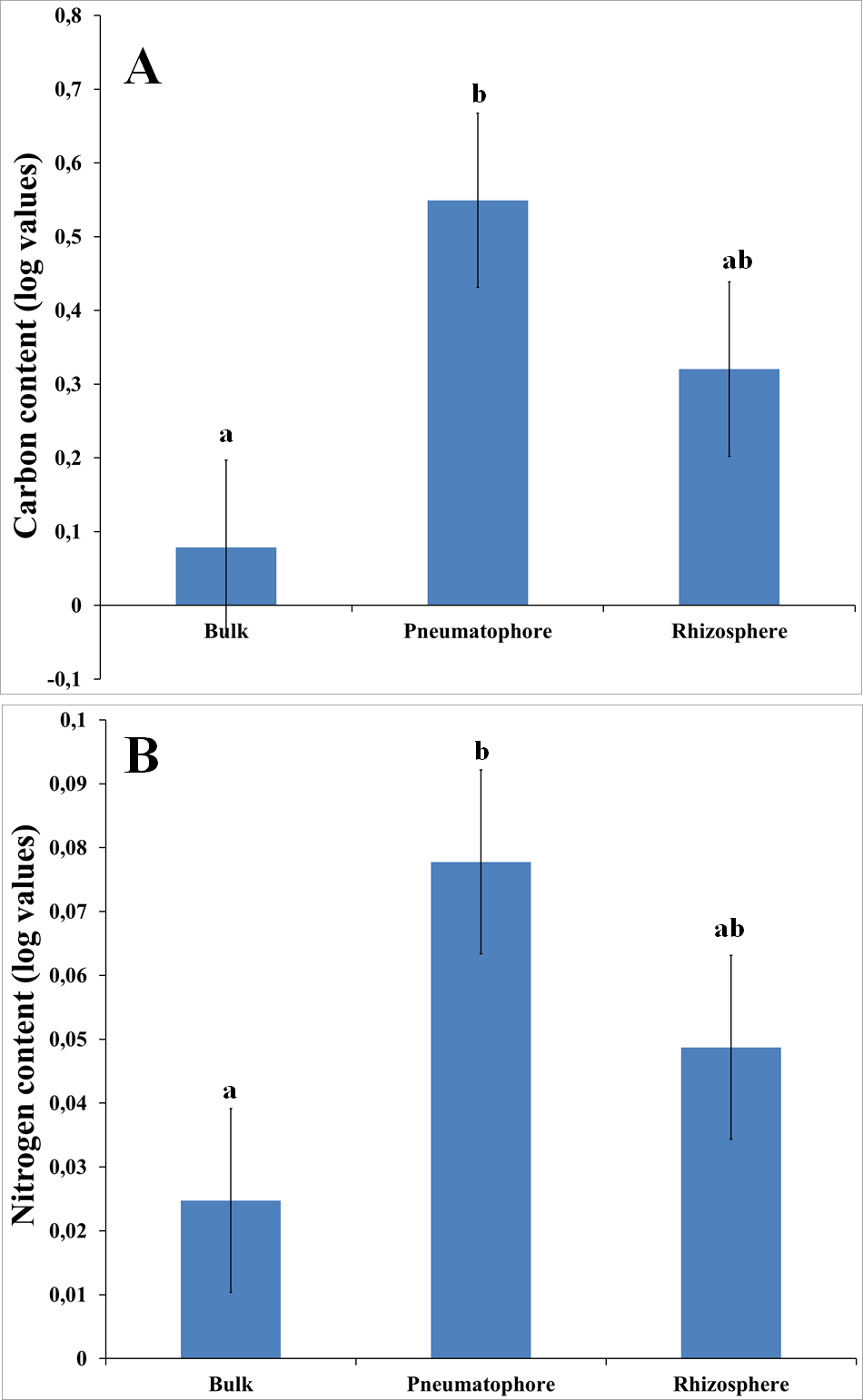


Supplementary figure 2. carbon (A), and nitrogen (B) content of bulk, pneumatophore and rhizosphere soil compartments. The different alphabet denotes significance at 0.05 (Bonferroni correction).


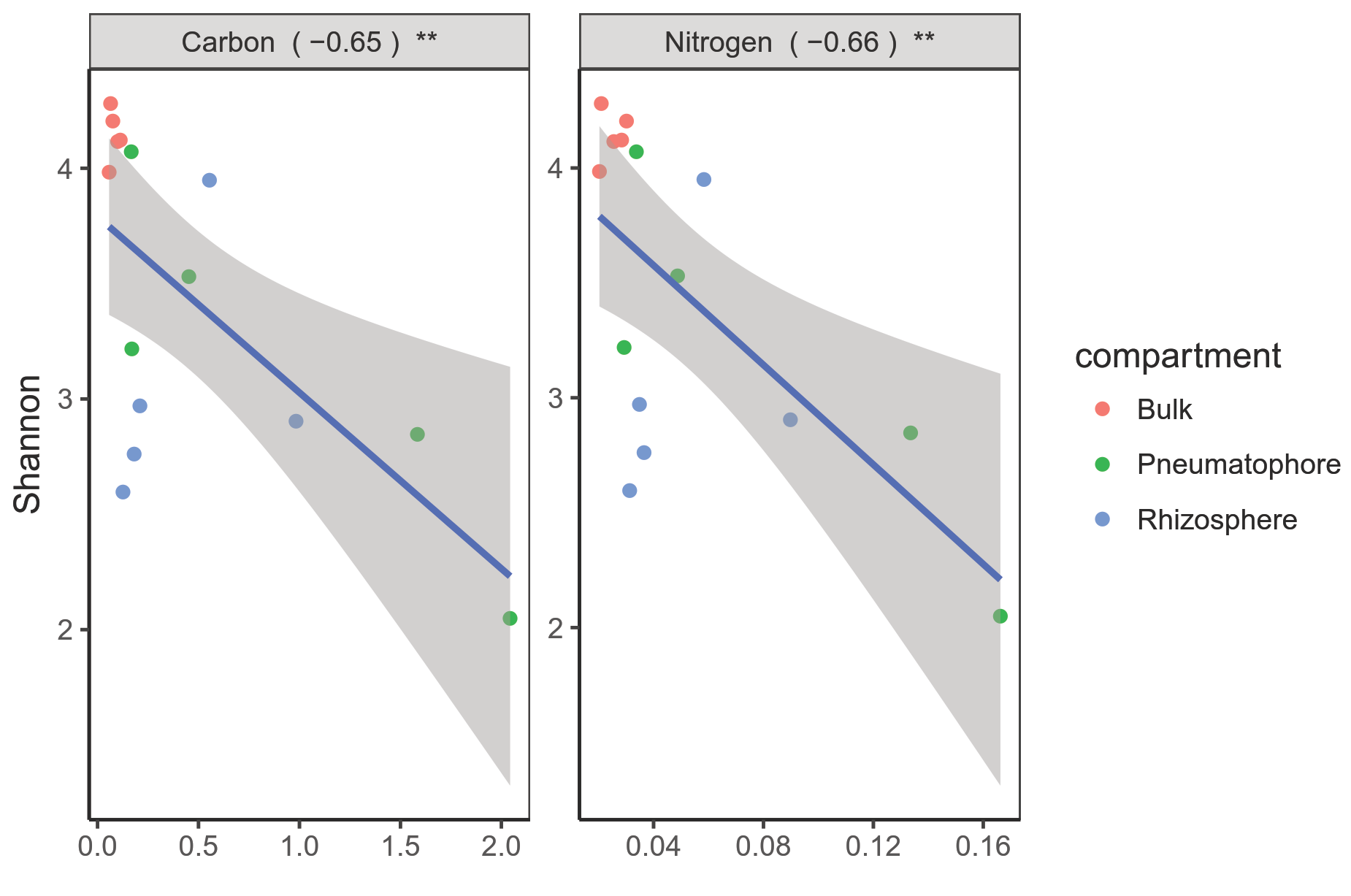


Supplementary figure 3. Correlation between Shannon diversity index and carbon or nitrogen content of different (bulk, pneumatophore and rhizosphere) soil compartments. Asterisks indicate the significant correlation at the 0.01 level (2-tailed).

| **Sample** | **Number sequences before the removal of OTUs with sequences less than five** | **Number sequences after the removal of OTUs with sequences less than five** | **Good’s coverage** |
| --- | --- | --- | --- |
| Bulk 1 | 3981 | 3886 | 0.984122 |
| Bulk 2 | 3902 | 3765 | 0.986143 |
| Bulk 3 | 3965 | 3842 | 0.986143 |
| Bulk 4 | 4790 | 4651 | 0.990185 |
| Bulk 5 | 3551 | 3464 | 0.980370 |
| Pneumatophore 1 | 11948 | 11700 | 0.971420 |
| Pneumatophore 2 | 10130 | 9880 | 0.975173 |
| Pneumatophore 3 | 14488 | 14195 | 0.978637 |
| Pneumatophore 4 | 7320 | 7099 | 0.984122 |
| Pneumatophore 5 | 13409 | 13206 | 0.980081 |
| Rhizosphere 1 | 6444 | 6296 | 0.983256 |
| Rhizosphere 2 | 6411 | 6224 | 0.995670 |
| Rhizosphere 3 | 7859 | 7681 | 0.983256 |
| Rhizosphere 4 | 12321 | 12102 | 0.984700 |
| Rhizosphere 5 | 7149 | 7030 | 0.982968 |
| Total | 117668 | 115021 |  |

Supplementary table 1. The number good quality sequences per sample before and after the removal of rare OTUs. The good’s coverage is calculated after the removal of rare OTUs.

| **Sources of variation** |  | **Sum of Squares** | **Degrees of freedom** | **Mean Square** | **F** | **p-value** |
| --- | --- | --- | --- | --- | --- | --- |
| Observed OTU richness | Between Groups | 8881,2 | 2 | 4440,6 | 6,9872 | 0,00972 |
|  | Within Groups | 7626,4 | 12 | 635,5333333 |  |  |
|  | Total | 16507,6 | 14 |  |  |  |
| Shannon diversity index | Between Groups | 3,7161147 | 2 | 1,85805736 | 6,41155 | 0,01276 |
|  | Within Groups | 3,477584 | 12 | 0,28979867 |  |  |
|  | Total | 7,1936988 | 14 |  |  |  |

Supplementary table 2. One way ANOVA (compartment as fixed factor) for observed OTU richness and Shannon diversity index of fungal OTUs in different (bulk, pneumatophore and rhizosphere) soil compartments.

| **Source of variation** | **Degrees of freedom** | **Sum of Squares** | **Mean Square** | **Pseudo-F** | **P(MC)** |
| --- | --- | --- | --- | --- | --- |
| compartment | 2 | 16772 | 8385.8 | 4.1215 | 0.001 |
| Residuals | 12 | 24416 | 2034.7 |  |  |
| Total | 14 | 41187 |  |  |  |

Supplementary table 3. Global PERMANOVA (compartment as fixed factor) for fungal community composition in different (bulk, pneumatophore and rhizosphere) soil compartments.

| 1. **Bulk Vs rhizosphere** | | | | | | |
| --- | --- | --- | --- | --- | --- | --- |
| **Source of variation** | **Degrees of freedom** | | **Sum of Squares** | **Mean Square** | **Pseudo-F** | **P(MC)** |
| compartment | 1 | | 11155 | 11155 | 5.5794 | 0.003 |
| Residuals | 8 | | 15994 | 1999.3 |  |  |
| Total | 9 | | 27149 |  |  |  |
| 1. **Bulk Vs pneumatophore** | | | | | | |
| **Source of variation** | | **Degrees of freedom** | **Sum of Squares** | **Mean Square** | **Pseudo-F** | **P(MC)** |
| compartment | | 1 | 10175 | 10175 | 6.8613 | 0.001 |
| Residuals | | 8 | 11863 | 1482.9 |  |  |
| Total | | 9 | 22038 |  |  |  |
| 1. **Rhizosphere Vs pneumatophore** | | | | | | |
| **Source of variation** | | **Degrees of freedom** | **Sum of Squares** | **Mean Square** | **Pseudo-F** | **P(MC)** |
| compartment | | 1 | 3828.2 | 3828.2 | 1.4601 | 0.197 |
| Residuals | | 8 | 20974 | 2621.8 |  |  |
| Total | | 9 | 24803 |  |  |  |

Supplementary table 4. Pairwise PERMANOVA (Compartment as fixed factor) for fungal community composition in different combinations (A) bulk soil Vs rhizosphere, (B) bulk soil Vs pneumatophore, and (C) rhizosphere Vs pneumatophore soil compartments.

1. Bulk Vs rhizosphere

1. Bulk Vs pneumatophore

1. Rhizosphere Vs pneumatophore

Supplementary table 5. The relative abundance of significantly (p<0.05) different fungal OTUs in different combinations of soil (A. bulk Vs rhizosphere, B. bulk Vs pneumatophore, and C. pneumatophore Vs rhizosphere) compartments.

| **Source of variation** | **Pearson correlation** | | | **Pearson correlation** | |
| --- | --- | --- | --- | --- | --- |
|  | **Shannon diversity index** | | **p-value** | **Obseverd OTU Richness** | **p-value** |
| Carbon | -0.648** | 0.009 | | 0.016 | 0.954 |
| Nitrogen | -0.660** | 0.007 | | 0.005 | 0.987 |
| ** Correlation is significant at the 0.01 level (2-tailed). | | | | | |

Supplementary table 6. Pearson correlation between Shannon diversity index or Observed OTU richness and carbon or nitrogen content of different (bulk, pneumatophore and rhizosphere) soil compartments.
